# Formulation-by-Design of Efinaconazole Spanlastic Nanovesicles for Transungual Delivery Using Statistical Risk Management and Multivariate Analytical Techniques

**DOI:** 10.3390/pharmaceutics14071419

**Published:** 2022-07-06

**Authors:** Rashed M. Almuqbil, Nagaraja Sreeharsha, Anroop B. Nair

**Affiliations:** 1 Department of Pharmaceutical Sciences, College of Clinical Pharmacy, King Faisal University, Al-Ahsa 31982, Saudi Arabia; sharsha@kfu.edu.sa (N.S.); anair@kfu.edu.sa (A.B.N.); 2 Department of Pharmaceutics, Vidya Siri College of Pharmacy, Off Sarjapura Road, Bangalore 560035, India

**Keywords:** efinaconazole, spanlastics, nail delivery, transungual, QbD, Ishikawa fishbone diagram, risk management, multivariate analysis

## Abstract

As regulatory and technical landscapes for pharmaceutical formulation development are rapidly evolving, a risk-management approach using multivariate analysis is highly essential for designing a product with requisite critical quality attributes (CQA). Efinaconazole, a newly approved poorly water-soluble antifungal triazole drug has poor permeability. Spanlastics, new-generation surfactant nanovesicles, being fluidic, help improve the permeability of drugs. Therefore, we optimized efinaconazole spanlastics using the concepts of Formulation-by-Design (FbD) and explored the feasibility of transungual delivery for the management of onychomycosis. Using the Ishikawa fishbone diagram, the risk factors that may have an impact on the CQA of efinaconazole spanlastic vesicles were identified. Application of the Plackett–Burman experimental design facilitated the screening of eight different formulation and process parameters influencing particle size, transmittance, relative deformability, zeta potential, entrapment efficiency, and dissolution efficiency. With the help of Pareto charts, the three most significant factors were identified, viz., vesicle builder (Span), edge activator (Tween), and mixing time. The levels of these three critical variables were optimized by FbD to reduce the particle size and maximize the transparency, relative deformability, encapsulation efficiency, and dissolution efficiency of efinaconazole spanlastic nanovesicles. Bayesian and Lenth’s analysis and mathematical modeling of the experimental data helped to quantify the critical formulation attributes required for getting the formulation with optimum quality features. The optimized efinaconazole-loaded spanlastic vesicles had a particle size of 197 nm, transparency of 91%, relative deformability of 12.5 min, and dissolution efficiency of 81.23%. The spanlastic formulation was incorporated into a gel and explored ex vivo for transungual delivery. This explorative study provides an example of the application of principles of risk management, statistical multivariate analysis, and the FbD approach in developing efinaconazole spanlastic nanovesicles.

## 1. Introduction

Onychomycosis (tinea unguium) is a fungal infection of the nails of the toe or fingers. It usually affects all the components of the nail unit (matrix, nail-bed, or nail-plate) [1]. It usually causes pain, discomfort, and disfigurement, producing certain limitations for physical and occupational activities that may adversely affect quality of life [2,3]. Topical therapy is at the forefront in treating nail ailments—especially onychomycosis, due to the perceived local effects—that circumvent systemic adverse events, improve patient compliance, and help reduce the treatment cost of therapy [4,5].

However, topical treatment of onychomycosis is challenging because locally applied drugs show poor permeability across the nail plate and most antifungal drugs have poor solubility and permeability [6]. The physiochemical properties of the nail are evidenced through various experiments indicating that the architecture and composition of the nail behave more like a hydrophilic gel membrane [7,8,9]. Efinaconazole, the recently approved potent triazole drug, inhibits the fungal ergosterol biosynthesis pathway and exhibits potent antifungal activity. However, poor solubility and permeability across nail plates limit its clinical effectiveness [10,11].

Topically applied systems with nanosized drugs offer the benefits of improved transungual penetration with minimal systemic adverse effects over conventional drug-delivery systems [12,13,14]. Spanlastic is novel self-assembly nanovesicles made from surfactants that offer an ultra-deformable delivery system. These particles efficiently accommodate poorly water-soluble drugs and show improved encapsulation and, therefore, were selected for exploring the improvement in transungual penetration of efinaconazole. The development of a robust nanotherapeutic system is a multi-pronged process that necessitates the careful and rational selection of variables of formulation and process [15,16]. However, the combined use of good-quality risk-management tools, screening, and experimental designs for optimization has not yet been explored for transungual spanlastics.

Rapid development in the field of nanoparticles and the regulatory quality initiatives, make it necessary to implement a holistic Formulation-by-Design (FbD) approach [17]. FbD involves controlled design and thorough analysis of a complicated process and product that are affected by many variables. Use of appropriate experimental designs, accurate identification of design and control space, identification of critical quality attributes (CQAs), critical formulation attributes (CFAs), and critical process parameters (CPPs) help achieve the goal of FbD. The present study is the first-ever successful application of the Ishikawa fishbone diagram, Plackett–Burman screening design followed by a full factorial design involving three factors at two levels for the development of efinaconazole-loaded spanlastic vesicles. The study further illustrated the implementation of FbD for validating the mathematical prediction models and identification of a formulation with optimum CQAs.

## 2. Materials and Methods

### 2.1. Materials

Efinaconazole was purchased from Cayman Chemicals (Mumbai, Maharashtra, India). Carbopol 934 was a gift sample from Lubrizol Advanced Materials India Ltd. (Mumbai, Maharashtra, India). Acetonitrile, Tween 80, Spans (60 and 65), sodium deoxycholate, phosphoric acid, and sodium phosphate were purchased from Merck Chemicals (Mumbai, Maharashtra, India). Water and all other chemicals and reagents were of analytical grade and used without any further purification.

### 2.2. Preparation of Spanlastic Nanovesicles Loaded with Efinaconazole

Efinaconazole-loaded spanlastic nanovesicles were prepared using ethanol injection method using vesicle builder (Span) and edge activator, either Tween 80 or sodium deoxycholate, at different concentration levels. Briefly, vesicle builder and efinaconazole were dissolved in ethanol. The alcoholic solution of efinaconazole was then injected slowly (1 mL/min) into preheated (70 ℃) aqueous phase (containing edge activator), with stirring. The mixture was stirred continuously at 70 ℃ and, subsequently, cooled at 5 °C, till further investigation. Various formulation and process parameters were screened and optimized.

### 2.3. Characterization of Efinaconazole Spanlastics

#### 2.3.1. Particle Size and Zeta Potential Measurement

Efinaconazole spanlastics were characterized for particles after being suitably diluted with double distilled water. The particle size using a nano-size analyzer (Nanophox, Sympatec India Pvt. Ltd., Mumbai, India) at ambient temperature [18]. Zeta potential was measured on Delsa Nano C, Beckman Coulter zeta meter (LabIndia, Mumbai, India) [19].

#### 2.3.2. Relative Deformability and Transmittance

Relative deformability, an indicator of elasticity of vesicles, was measured by extruding the formulation through a polycarbonate filter (pore size 220 nm, Merck Millipore, Merck Ltd., Mumbai, India), at a constant pressure of 0.17 MPa [20]. The relative deformability was measured in terms of the time required for extrusion of 10 mL of formulation. The experiment was repeated, and the results are expressed as the mean of six determinations ± SD [21]. To analyze the transparency, transmittance (%) of the undiluted spanlastics was measured at 600 nm using (UV-Vis Spectrophotometer, Systronics, Ahmedabad, India).

#### 2.3.3. Estimation of Efinaconazole Content

Encapsulation efficiency was analyzed by the validated RP-HPLC method reported earlier with slight modification [22,23]. The RP-HPLC analysis was carried out at 22 °C (Jasco PU-2080 Plus, Intelligent HPLC pump, Japan, Detector of Jasco 2075, Intelligent UV–vis detector, Jasco, Tokyo, Japan, and RP-C18 column of Agilent, 250 × 4.6 mm, 5 μm particle size, Technologies, Mumbai, India). 20 μL of each sample was injected after suitable dilution with mobile phase and detected at 210 nm. The mobile phase consisted of phosphate buffer pH 5.5 and acetonitrile (25:75), and an isocratic flow of 1 mL/min was used [23].

#### 2.3.4. Dissolution Efficiency of Efinaconazole-Loaded Spanlastics

The release of efinaconazole was studied by suspending efinaconazole-loaded spanlastics in a Float-A-Lyzer (G2, Spectrum, Repligen, MA, USA) in phosphate buffer saline pH 7.4. The tests were performed at 37 °C (n = 6) by introducing the dialyzers into 100 mL release media stirred at 100 rpm [24]. Efinaconazole released at different time intervals up to 4 h was analyzed using the above-mentioned method by withdrawing 0.5 mL of release media. The volume of release media was maintained at 100 mL by replacing an equal volume of release media immediately after sampling. Release media samples were filtered through 0.22 μ PVDF filters (Millex-VV, 13 mm, Merck, Mumbai, India) and analyzed after appropriate dilution with a mobile phase of the RP-HPLC method. Dissolution efficiency at the end of 4 h was calculated. It is the % of the ratio of the area under dissolution curve from 0–4 h to the area of the rectangle in the same period considering 100% dissolution. The areas under dissolution curves were calculated using the Trapezoidal rule [15].

### 2.4. Identification of Cause–Effect Relationship Using Ishikawa Fishbone Diagram

For risk-analysis operation to configure the cause–effect relationship between CFAs and CPPs and the CQAs, the Ishikawa fishbone diagram was constructed [25,26]. In the formulation of spanlastics, particle size, zeta potential, entrapment efficiency, relative deformability, transmittance, and dissolution efficiency were identified as key CQAs that influence the biopharmaceutical properties of spanlastics, based on the literature available and preliminary experimentation [17,27].

### 2.5. Screening of Risk Assessment of Several Variables 

Plackett–Burman screening design (PBSD), with 12 formulation runs for eight factors, was employed for screening of identified high-risk CFAs and CPPs that can influence the CQAs. The levels of selected CFAs and CPPs are listed in Table 1. Based on preliminary trial experiments, lower (−1) and higher (+1) levels for all eight independent variables were decided. The dependent responses selected as CQAs were particle size (Y_1_, nm), zeta potential (Y_2_, mV), encapsulation efficiency (Y_3_, %), transmittance (Y_4_, %), and dissolution efficiency (Y_5_, %). Experimental design, randomization of experiments, and statistical analysis of the data were carried out using NEMRODW software (LPRAI SARL, Marseille, France). Each batch of the formulation was prepared six times and mean values were recorded (Table 2). The significance of the PBSD and coefficients for all eight independent variables were generated using multiple regression analysis and the data were analyzed using analysis of variance (ANOVA). The influence of each parameter was estimated using Pareto charts. The charts were plotted for individual contribution (%) as well as for cumulative contribution (%) using normalized squares.

### 2.6. Optimization of Efinaconazole Spanlastics Using Full Factorial Design

Post-screening of influencing factors, full factorial design (2^3^) was applied using the CFAs and CPP identified using PBSD. This was done to achieve an optimum formulation and process, and also to identify interaction among the selected parameters if any. The five factors showing the statistically insignificant effect on the CQAs were kept unchanged (Table 3). The remaining three CFAs, namely, amount of vesicle builder—Span 60 (X_1_), amount of edge activator—Tween 80 (X_2_), and sonication time (X_3_), were optimized using FbD, wherein the high and low levels were kept at the same level as in screening design (Table 3). NEMRODW software (LPRAI SARL, Marseille, France) was used to generate and evaluate the experimental design. The CQAs studied are listed in Table 3. The levels of the selected three CFAs and CPPs were changed during experimentation and, subsequently, CQAs studied were mean particle size of spanlastics (Y_1_, nm), relative deformability (Y_2_, %), transmittance (Y_3_, %), and dissolution efficiency (Y_4_, %). All the batches of FbD (FD1-FD8) and their results data are presented in Table 4. Polynomial equations were generated to determine the relationship between CFAs/CPP and CQAs by applying ANOVA at the 5% significance level. The *p*-value is <0.05 indicates that the model is significant. Lenth’s method and Bayesian analysis were used to check the impact of CFAs and the interaction between them on each of the CQA.

### 2.7. Validation of Experimental Design and Optimization of Formulation

The optimum composition was identified by putting constraints on independent variables in the mathematical equation to minimize the particle size and relative deformability and maximize transmittance and dissolution efficiency. One optimum formulation composition (A) and two random formulation compositions (B and C) were prepared and analyzed. Formulations corresponding to these three batches were prepared and evaluated for the desired CQAs (Y_1_–Y_4_). The results were evaluated for prediction error (% bias).

### 2.8. Efinaconazole Spanlastic Optimized Formulation and Converting into Gel

#### 2.8.1. Topography Using Electron Microscopy

For scanning electron microscopy (SEM) in cryo mode, the spanlastic formulation, blank carbopol gel, and efinaconazole-loaded spanlastic dispersed in carbopol gel were frozen in liquid nitrogen, followed by coating with platinum. Samples were viewed in FEG-SEM (Quorum Technologies, Laughton, East Sussex, UK) [28,29].

#### 2.8.2. Ex Vivo Permeation through Bovine Hoof Membranes

A bovine hoof membrane was obtained from the local slaughterhouse. They were hydrated in phosphate buffer saline pH 7.4 overnight. The hydrated bovine hoof membrane was carefully mounted over the receptor chamber of vertical static jacketed Franz diffusion cells (effective diffusion area 3.14 cm^2^, the thickness of bovine hoof membrane 400 µm) [30,31]. The TEER value was measured before and after the experiment to ensure the intactness of the membrane and was found to be 1420 ± 82 Ω·cm^2^ and 1460 ± 76 Ω·cm^2^, before and after the experiment, respectively.

The donor chamber was filled with phosphate buffer saline pH 7.4 (7 mL) [32] previously filtered through a 0.22 μm PVDF filter and maintained at 32 ± 0.5 °C. The test formulations were loaded into the donor chamber and the efinaconazole permeated through the hoof membrane was analyzed by withdrawing samples from the receptor chamber at predetermined time intervals (30 min, 1, 2, 3, 4, 6, 8, and 12 h). The volume of the receptor chamber was maintained by replacing the equal volume of release media. The samples withdrawn were filtered and analyzed by the RP-HPLC method described above. The results were expressed graphically as cumulative efinaconazole permeated versus time (h). Every formulation was tested in multiples of six (n = 6) [28].

## 3. Results and Discussion

### 3.1. Cause–Effect Relationship for Efinaconazole-Loaded Spanlastics Using Ishikawa Fishbone Diagram

FbD approach needs the identification of independent variables in formulation and process that can have an impact on the performance of the product. Ishikawa fishbone is a systematic pictorial qualitative exploration of the main causative factors and their sub-causes influencing the quality of the product. Thus, it is a tool to represent the cause–effect relationship in a simplified way [17,25].

**Figure 1:**
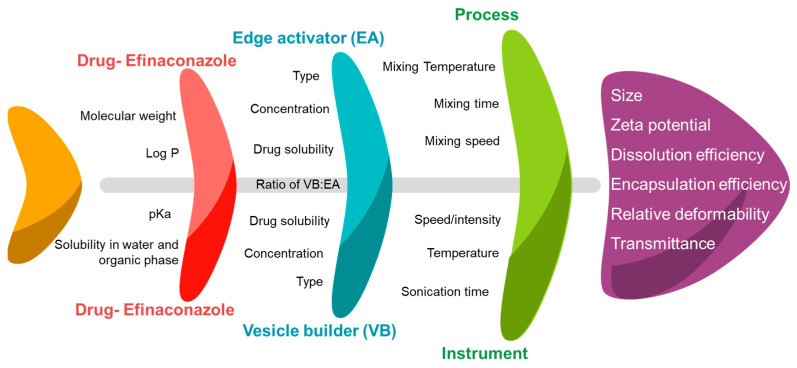
An Ishikawa fishbone diagram displaying the cause-effect relationship of several independent CFAs and CPPs that can affect the critical quality parameters of efinaconazole spanlastics.

Ishikawa diagram was constructed to summarize all the independent variables of formulation and the manufacturing process that may affect the quality features of efinaconazole spanlastic nanovesicles and is presented in Figure 1.

Compared to other nano-colloidal systems, spanlastics are surfactant-based, relatively deformable, and flexible carriers than can easily cross the tough biological membranes [16,33]. Factors such as type of vesicle builder, edge activator, the proportion of organic to aqueous phase along with a technique for preparation that involves speed, time, temperature, etc., affect the properties of spanlastics. Ethanol was selected as the organic phase as it solubilizes the selected drug and the vesicle builder. Additionally, rapid evaporation of the ethanol reduces the overall processing time. After a few preliminary feasibility trials, eight different variables were selected for screening and further optimization. These prioritized CFAs and CPPs were as follows: type of vesicle builder (X_1_), type of edge activator (X_2_), mixing time (X_3,_ min), amount of organic phase (X_4_, mL), sonication time (X_5_, min), amount of efinaconazole (X_6,_ mg), mixing speed (X_7_, rpm), and volume of aqueous phase (X_8_, mL).

### 3.2. Risk Assessment Screening Using Pareto Charts

Statistical model-based PBSD is often used to screen a large number of variables as it involves fewer experimental runs [34]. Nanospanlastics received great attention in recent years as potential nanocarriers as they exhibit considerably higher elasticity compared to other nanoparticulate systems [35] and also have high permeability through tough biological barriers, such as nail plate [13]. Spanlastic nanovesicles are composed of nonionic surfactants which act as vesicle builders and edge activators that decrease the interfacial tension and impart fluidity and deformability to vesicles required for easy permeation [35].

Nanosize is the main feature of the product responsible for improving the encapsulation of poorly water-soluble drugs and also for permeability across tough biological barriers. Therefore, particle size was considered as the CQA. Zeta potential, the electrical charge of the particles, contributes to the stability of the colloidal system. If the zeta potential is between (–) 30 mV and (+) 30 mV, the colloidal system may show instability such as flocculation or aggregation promoted due to Van der Waals attractions. Many times, zeta potential also gives a rough estimate of the location of charged drugs whether entrapped or adsorbed on the surface of particles [36]. Therefore, the zeta potential was considered the second CQA. Flexibility and deformability are the critical aspects of elastic spanlastic nanovesicles that make them unique from other colloidal carriers [37]. The use of an appropriate edge activator makes the particles elastic enough to squeeze through pores with a size smaller than their diameters. Therefore, relative deformability was selected as the third CQA. Low turbidity products are potentially advantageous for topical application. The turbidity of the product can be easily analyzed by measuring the transmittance of the product [38]. Therefore, transmittance was selected as the fourth CQA. Nanotechnology offers several benefits, such as enhanced retention and controlled release of the drug, reduction of toxicity issues, and site-specific drug delivery [39]. Additionally, nanovesicular systems also increase drug loading, especially for water-insoluble drugs. Therefore, encapsulation efficiency was selected as the fifth CQA in PBSD.

**Figure 2:**
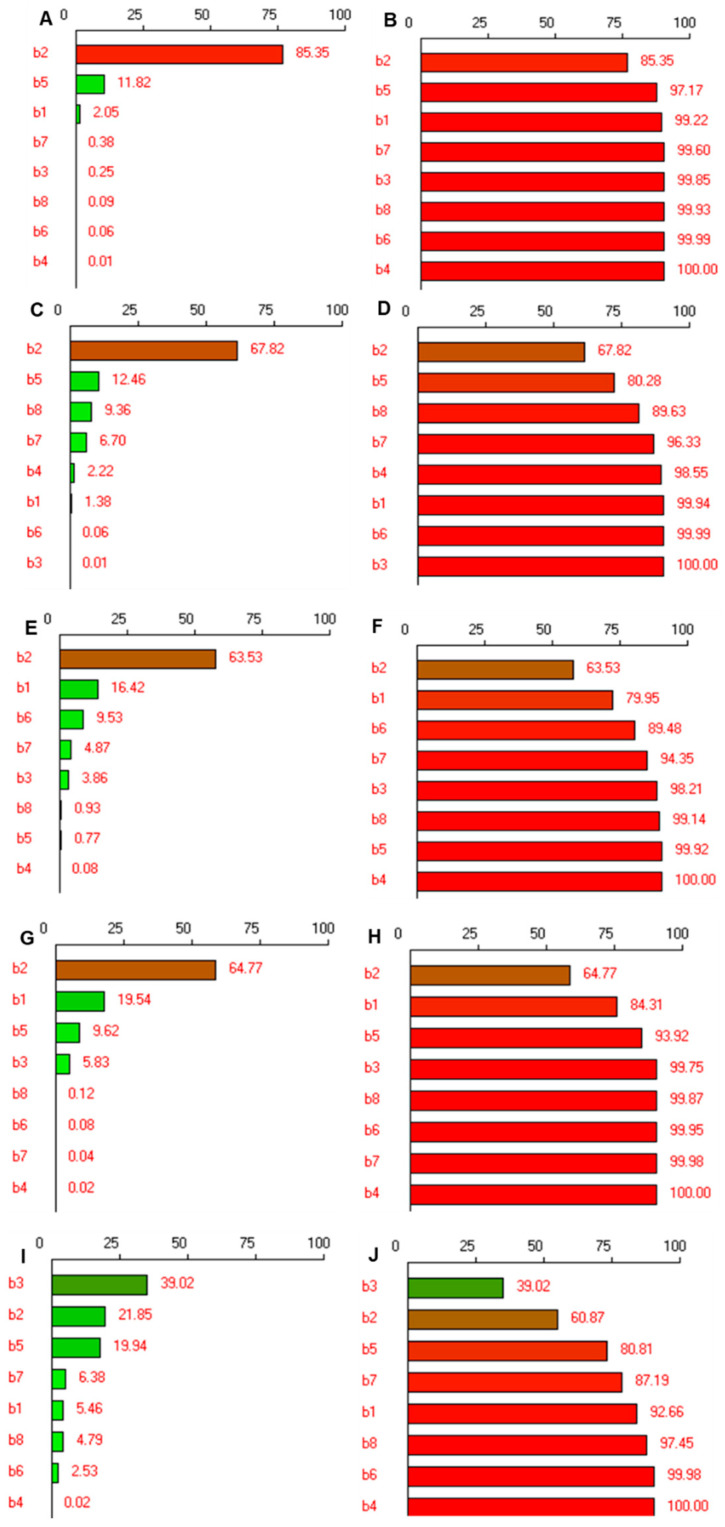
Pareto charts portraying the effect of selected critical formulation and process parameters on characteristics of efinaconazole spanlastic formulation. (**A**,**C**,**E**,**G**,**I**) and (**B**,**D**,**F**,**H**,**J**) represent individual and cumulative contribution (normalized squares, %) for particle size (Y_1_), zeta potential (Y_2_), relative deformability (Y_3_), transmittance (Y_4_), and encapsulation efficiency (Y_5_), respectively. b1 to b8 represent the values of coefficients for the selected eight factors in the experimental design.

Individual contribution (normalized squares, %) and cumulative contribution (%) for particle size, zeta potential, relative deformability, transmittance, and encapsulation efficiency are presented in the form of Pareto charts in Figure 2.

ANOVA-derived related statistical parameters, namely, model *p*-value, coefficient of regression, F-ratio, etc., were analyzed (Table 5).

The estimated coefficients are abbreviated as b1–b8 for X_1_–X_8_ obtained through regression analysis for the selected CQAs; Y_1_–Y_5_. For particle size Y_1_, the coefficient of regression, i.e., r^2^ is 0.99, which signifies that 99% of the changes in the particle size could be explained by the model and confirms the goodness of fit of the model. Low probability value *p* = 0.0012 and Fisher test critical value (F-ratio) greater than the theoretical confirm the high significance of the regression model at a confidence level of 95% (Table 5). As observed from Figure 2A, the type of edge activator is the most significant factor responsible for the change in the particle size with 85.35% contribution and also the highest magnitude of coefficient b2 = −194.42 with a *p*-value of 0.001 (Table 5). The minus sign of the coefficient indicates that a higher level of X_2_, i.e., Tween 80, causes a most significant decrease in particles compared to sodium deoxycholate. Therefore, Tween 80 was selected for further optimization. Apart from the type of edge activator, sonication time (X_5_) also influences the size of spanlastics, however, the contribution is 11.82% (Figure 2A). The negative sign of the coefficient of b5, −73.08, suggests that an increase in sonication time reduces the particle size. The third significant factor affecting particle size was vesicle builder. In the cumulative Pareto chart (Figure 2B), the contribution by these two independent variables in reducing the particle size of efinaconazole spanlastics is 97.17%. Additionally, Span 65 also contributed significantly (*p* = 0.023, Table 5) to the reduction in particle size as observed from the negative coefficient of b1, −30.42 (Table 5), with an individual contribution of 2.05%, as observed in Pareto charts (Figure 2A,B). This difference in influence on the particle size of spanlastics may be due to the difference in phase transition temperature (Span 60 = 53 °C and Span 65 = 14 °C) [13,40].

For zeta potential, although the model was significant (*p* = 0.008) and there was a good correlation as observed from r^2^ = 0.90, none of the independent variables was significantly active on the surface charge of particles (Table 5). As observed from the Pareto chart, Tween 80 was active in modifying the surface charge, however, was not having a significant contribution (*p* = 0.23). For good physical stability by steric repulsion of nano colloidal systems, it is expected to have zeta potential above (+)30 or below (−)30 mV [36]. However, spanlastics are made from non-ionic surfactants, therefore, a higher interfacial charge is not achieved. The small magnitude of negative charge is generally due to the ionization of free fatty acids present in the surfactants. The Pareto charts (Figure 2C,D) show the contribution of Tween 80 for the surface charge, but being a non-significant parameter, hence, it cannot be considered as a CFA for influencing the zeta potential. Therefore, the zeta potential was not considered as the CQA later for optimizing the formulation using full factorial design.

Highly deformable flexible systems overcome the limitation of poor permeability of drugs through tough biological barriers [41]. Therefore, we measured the relative deformability and observed that type of edge modifier, vesicle builder, amount of efinaconazole, and the mixing speed were the significant factors affecting the flexibility of efinaconazole spanlastics in the present study (94.35% contribution, Figure 2E,F). Tween 80 and Span 60 were making the vesicles more flexible, as can be noted from the negative coefficient values of b2 and b1, −11.02 and −0.56 with *p* values of 0.001 and 0.009, respectively. (Table 5) These two factors together contributed to 79.9% influence on deformability of vesicles (Figure 2F). The difference in chemical structures of edge activators changes the flexibility of vesicles. Highly flexible and non-bulky hydrocarbon chains of Tween 80 made the particles flexible compared to steroid-like structures of sodium deoxycholate [21]. In the case of the vesicle builder, Span 65 has a lower HLB of 2.1 compared to Span 60 (HLB 4.7) [42]. This indicates more hydrophilicity of Span 60 with increased mobility of the hydrocarbon chains in presence of an aqueous phase that facilitates conformational changes that are energetically stimulated rotations to relieve the stress [43].

The transparency of the product greatly influences patient acceptance in the case of topically applied products. Therefore, transmittance was measured as the indicator of transparency. The higher the transmittance, the higher the clarity or transparency of the nanovesicle formulation. In the present study, the edge activator was the only factor significantly acting on the clarity of the product (*p* = 0.011, Table 5) with a contribution of 65.77% (Figure 2G,H).

Encapsulation was found to be significantly influenced by only the surfactants and time (Table 5) with an individual contribution of vesicle builder, edge activator, mixing time, and sonication time as 39.02%, 21.85%, 19.94%, and 6.38% (Figure 2I), respectively, together making a contribution of 87.19% (Figure 2J). Being a water-insoluble drug, efinaconazole encapsulation was found to be increased due to the presence of surfactants. The chain length and size of the hydrophilic head group of the nonionic surfactant have a strong influence on the encapsulation of the drug. Nonionic surfactants with stearyl chains and Tween surfactants with a long alkyl chain and a large hydrophilic moiety exhibit high entrapment efficiencies [44]. The use of sonication during the process of manufacture of vesicles from the drug and carrier mixture increases the rate of mass transfer that could intensify the drug dissolution to increase the solubility and, in turn, the encapsulation [45].

As observed from this PBSD and the corresponding analysis, it was found that zeta potential, usually a significant parameter determining the stability of colloidal carriers, was not affected by the selected CFAs and CPPs. Tween 80, Span 60, and the sonication time were the most influential factors and, thus, were selected for further optimization.

### 3.3. Full Factorial Experimental Design (2^3^, 3 Factors at Two Levels)

Amount of vesicle builder—Span 60 (X_1_), amount of edge activator—Tween 80 (X_2_), and sonication time (X_3_) were identified as the CFAs that impact the quality of efinaconazole-loaded spanlastics. For a systematic exploration of these factors on the efinaconazole-loaded spanlastics, the number of individual factors was varied according to 2^3^ full factorial designs, and the effects of these alterations in the factors were studied. The data were fitted to the first-order polynomial regression equation, as mentioned below:

Y= b0 + b1X_1_ + b2X_2_ + b3X_3_ + b12X_1_X_2_ + b13X_1_X_3_ + b23X_2_X_3_ + b123X_1_X_2_X_3_ (1)

where, b0: the arithmetic mean of all the quantitative outcomes of the experimental runs, b1, b2, and b3: the coefficients calculated from the experimental values of Y for X_1_, X_2_, and X_3_, Xp Xq: p and q can be any number from 1, 2, and 3 indicating interaction.

**Figure 3:**
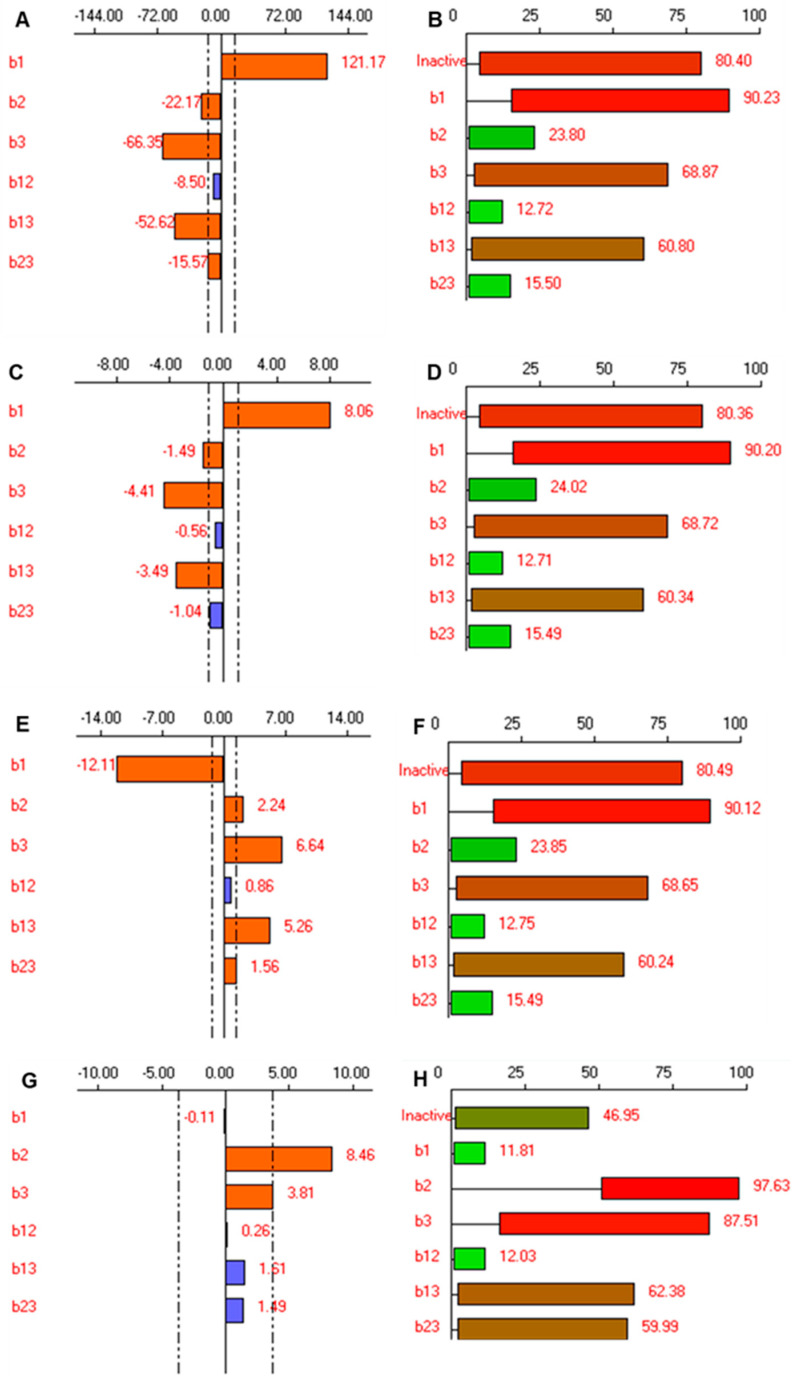
Lenth’s graphical analysis (**A**,**C**,**E**,**G**) and Bayesian analysis of the coefficients (**B**,**D**,**F**,**H**) indicate the probability of each CFA’s (X) activity for all the considered CQAs (Y), (**B**,**D**,**F**,**G**) for Y_1_ (particle size), (**D**) Y_2_ (relative deformability), (**F**) Y_3_ (transmittance) and (**H**) Y_4_ (dissolution efficiency), respectively.

One factor along with its coefficients indicates the effect of that particular factor while the cross-product of two or more factors along its coefficients represents the interaction term among these factors. The sign in front of the terms is indicative of the synergistic or antagonistic effect of the factors. The synergistic effect can be expected with the term having a positive sign while factors with a negative sign indicate an antagonistic effect on the outcome. Lenth’s graphical analysis was carried out to identify the optimum CFA level. Further, the graphical analysis also indicates the levels of the factors that are active in response. The bars corresponding to the active factors can be easily identified as these bars exceed the two dotted vertical reference lines. The two dotted reference lines represent the experimental variance (Figure 3A,C,E,G). The probability of each active effect is represented graphically as box plots and coefficients of Bayesian analysis were also calculated (Figure 3B,D,F,H) [46].

#### 3.3.1. Effect on the Particle Size of Spanlastics

The size of the spanlastics is an important CQA influencing the transungual permeability, biological fate, and efficacy of the spanlastics. [47] The efficacy and penetration of the nanovesicles are highly dependent upon the vesicle size. The smaller particle size ensures deeper penetration of the nanovesicles [13]. The nanosized spanlastics can easily cross the transungual barrier due to their easy penetration and small size. Thus, preparing spanlastics with low particle size to guarantee deeper penetration was an important goal for this study. The particle size of efinaconazole-loaded spanlastics ranged from 180.50 nm (FD7) to 602.20 nm (FD2) (Table 4). Independent factors affecting particle size significantly (*p* < 0.05) were the amount of vesicle builder—Span 60 (X_1_), amount of edge activator—Tween 80 (X_2_), and sonication time (X_3_) (Table 6).

The Equation for particle size is:

Y_1_= 345.80+ 121.75 X_1_ − 22.175 X_2_ −66.35 X_3_ −8.50 X_1_X_2_ −52.625X_1_X_3_ − 15.575X_2_X_3_ (2)

An increase in the amount of Span 60 tends to increase the particle size, whereas the inverse is observed with an increase in the amount of Tween 80 as well as an increase in sonication time. The same is reflected in the equation as well. Further, ANOVA results revealed that increasing the concentration of edge activator (X_2_) and sonication time (X_3_) resulted in a significant reduction in the particle size. In the current study, Tween 80 is the selected edge activator that, owing to its lower bulkiness and unsaturation, guarantees the integration of edge activator into the nanovesicles and its enhanced chains bending, leading to small sized-particles [48]. Small particles obtained by increasing the amount of Tween 80 on the particle size could be attributed to the reduction of interfacial tension by increasing the surfactant concentration. Similar results have been reported in another study by Dora et al. wherein a greater amount of the edge activator reduced the surface tension, enabling particle partition and the formation of smaller nanovesicles of glibenclamide [49]. A similar effect of edge activator on the size of olanzapine transfersomal vesicles has been reported by another study [50].

Sonication of efinaconazole-loaded spanlastics for 5 min resulted in a significant decrease in the particle size of the vesicles. This could be attributed to the exposure of the particles to ultrasonic radiation, which results in the dispersion of the vesicles into smaller sizes. Similar results have been reported in several studies [50,51,52,53,54]. Similar effects of sonication time on particle size have been reported by Ngan et al. wherein a longer duration of ultrasonic radiation reduces the dispersion of the nanoemulsion droplets into smaller sizes [55].

The amount of Span 60 had a significant effect on particle size as observed from the positive coefficient of X_1_ in Equation (2) and significant bar length in Figure 3A. Span 60 is the lipidic vesicle builder with surfactant-like properties. An increase in particle size with an increase in the amount of Span 60 has also been reported earlier in several studies [13,56,57].

The coefficient value for the interaction terms X_1_X_2_ was statistically insignificant, however, the interaction terms X_1_X_3_, and X_2_X_3_ showed that the interaction between these factors was statistically significant (*p* < 0.05, Table 6). The negative sign of their coefficients (b13 and b23) showed an antagonistic effect on the particle size. Bayesian analysis of the coefficients (Figure 3B) shows that the effects b_1_, b_3_ and the interaction terms b_13_ are active, with probabilities of 90.23%, 68.87%, and 60.850%, respectively. The probability of insignificant effects, b_2_, b_12,_ and b_23_, was found to be considerably lower at 23.80%, 12.72%, and 15.50%, respectively.

#### 3.3.2. Effect on Relative Deformability of Spanlastics

Deformability is the critical aspect of elastic vesicles that distinguishes them from other conventional colloidal carriers in crossing biological membranes [50]. It is well known that the incorporation of edge activators into the vesicle bilayer is pertinent to the elasticity of spanlastic vesicles [58]. The elasticity of the spanlastic vesicles enables them to squeeze themselves through pores much smaller than their diameters under the influence of water gradient [59]. Table 4 illustrates that the relative deformability of the prepared nano-spanlastics ranged from 12.00 to 37.90 min. Figure 3C shows the effect of the three variables on the relative deformability of the efinaconazole-loaded spanlastics vesicles. Results revealed that independent factors, X_1_ (amount of Span 60), X_2_ (amount of Tween 80), and X_3_ (sonication time) exhibited significant effects on the relative deformability of the prepared vesicles (*p* < 0.05). The individual and interaction effects of the factors on the relative deformability can be explained by the following regression Equation:

Y_2_= 23.037+ 8.062 X_1_ −1.487 X_2_ − 4.413 X_3_ − 0.562 X_1_X_2_ − 3.488 X_1_X_3_ − 1.037X_2_X_3_ (3)

It can be noted that the formulae containing a high amount of both vesicle builder and edge activator when processed without sonication exhibited the highest relative deformability. This could be well explained because of the size of the spanlastic vesicles. Higher concentration of Span 60 results in large nanovesicles that are less elastic (FD4). A bulkier and less elastic spanlastic vesicle will face higher resistance to crossing through the membrane pore. As previously stated in section (particle size), Tween 80 (FD7) exhibited the least bulky structure, so it gave rise to vesicles of the highest elasticity. By inspection of the results, it is clear that the vesicle elasticity increased by increasing the amount of the Tween 80. This might be attributed to the fluidization of the vesicle bilayer produced by the high edge activator concentration. Increasing the sonication time also resulted in an improvement in the elasticity of the spanlastic vesicles, as indicated by the large negative coefficient of X_3_. The small size imparted by the sonication waves that results in small vesicle size seems to be contributing to the improved relative deformability of the vesicles. Bayesian analysis of the coefficients (Figure 3B) shows that the effects b_1_, b_3_ and the interaction terms b_13_ are active, with probabilities of 90.20%, 68.72%, and 60.30%, respectively. The probability of insignificant effects, b_2_, b_12,_ and b_23_, was found to be considerably lower, at 24.02%, 12.71%, and 15.49%, respectively.

#### 3.3.3. Effect on Transmittance

The clarity of the spanlastic formulation is an important parameter that reflects the character of the particles formed in the formulation. Spanlastic with a good number of vesicles appears to be turbid. A higher amount of edge activator results in the formation of mixed micelle or ruptured vesicles [21]. Table 4 illustrates that the clarity of the spanlastics as measured in terms of transmittance ranged from 49.8% to 92.0%. Figure 3E shows the effect of the three variables on the transmittance (%) of the efinaconazole-loaded spanlastics vesicles. Results revealed that independent factors, X_1_ (amount of Span 60), X_2_ (amount of Tween 80), and X_3_ (sonication time) exhibited significant effects on the transmittance of the prepared vesicles (*p* < 0.05). The individual and interaction effects of the factors on transmittance can be explained by the following regression Equation:

Y_3_= 75.462 − 12.112 X_1_ + 2.237 X_2_ + 6.637 X_3_ + 0.863 X_1_X_2_ + 5.5262 X_1_X_3_ + 1.562X_2_X_3_ (4)

As reflected in the equation, the amount of Span 60 has an inverse effect on the transmittance of the formulation while the amount of Tween 80 and sonication time has a direct relation with the transmittance. Interestingly, the interaction terms X_1_X_3_ and X_2_ X_3_ also significantly affect the transmittance of the spanlastic formulation (Table 6). Though, the effect of the interaction term X_1_X_3_ is prominent in comparison to the effect of X_2_X_3_. Further, Bayesian analysis of the coefficients (Figure 3F) shows that the effects b_1_, b_3_, and the interaction terms b_13_ are active, with probabilities of 90.12%, 68.65%, and 60.24%, respectively. The probability of insignificant effects, b_2_, b_12,_ and b_23_, was found to be considerably lower, at 23.85%, 12.75%, and 15.49%, respectively.

#### 3.3.4. Effect on Dissolution Efficiency

Dissolution efficiency is reflective of the drug-release pattern from the formulation. It is the area under the curve of the drug-release profile between defined time points [60]. The eight spanlastic formulations exhibited a drug-release profile ranging from 57.10% to 85.40%. Figure 3G shows the effect of the three variables on dissolution efficiency (%) of the drug-loaded spanlastic vesicles. Results revealed that independent factors, X_2_ (amount of Tween 80) and X_3_ (sonication time), exhibited significant effects on the dissolution efficiency of the prepared vesicles (*p* < 0.05). The individual and interaction effects of the factors on dissolution efficiency can be explained by the following regression Equation:

Y_4_= 69.587 − 0.112 X1 + 8.462 X_2_ + 3.812 X_3_ + 0.263 X_1_X_2_ + 1.613 X_1_X_3_ + 1.487 X_2_X_3_ (5)

The amount of Tween 80 has a prominent and significant direct correlation with the amount of drug release from the efinaconazole-loaded spanlastics. An increase in the amount of Tween 80 improves the dissolution efficiency as the increase in Tween 80 results in a smaller particle size. Also, the unsaturated alkyl chain of Tween 80 imparts the chain fluidity and permeability and, hence, aids in higher dissolution efficiency [16]. Similarly, an increase in sonication time results in smaller particle size and, hence, the large surface area, thereby resulting in higher dissolution efficiency. None of the interaction terms has a significant effect on the dissolution efficiency (Table 6). Further, Bayesian analysis of the coefficients (Figure 3H) shows that the effects b2 and b3 are active, with probabilities of 97.63% and 87.51%, respectively. The probability of insignificant effects, b_1_, b_12_, b_13,_ and b_23_, was found to be considerably lower, at 11.81%, 12.03%, 62.38%, and 59.99%, respectively.

### 3.4. Optimization and Validation of the Developed Mathematical Model

The polynomial equation for all four responses was used to optimize the composition of efinaconazole-loaded spanlastics. In each set, the optimal composition was obtained after fixing the desired CFAs. Accordingly, it is desirable to minimize particle size and relative deformability while maximizing transmittance and dissolution efficiency to obtain efinaconazole-loaded spanlastics with improved biopharmaceutical characteristics. The desirability was kept at more than 0.9. For the efinaconazole-loaded spanlastics, the optimization constraints applied were Y_1_ (<200 nm), Y_2_ (<13 min), Y_3_ (>90%), and Y_4_ (>80%).

Detailed analysis of the data obtained from the factorial design is used to find the optimum levels of each factor. The optimal calculated levels are as follows:

Amount of vesicle builder − Span 60 (X_1_) = 105 mg/mL

Amount of edge activator − Tween 80 (X_2_) = 100 mg/mL

Sonication time (X_3_) = 5 min

The efinaconazole-loaded spanlastics prepared with the above-mentioned optimized CFAs levels exhibit Y_1Experimental_ as 197.00 nm (Y_1Predicted_, 196.23 nm; bias 0.39%), Y_2Experimental_ as 12.50 min (Y_2predicted_, 13.09 min; bias, −4.51%), Y_3Experimental_ as 91.00% (Y_3Predicted_, 90.19%; bias, 0.90%), and Y_4Experimental_ as 81.23% (Y_3Predicted_, 82.03; bias, −0.97%), as shown in Table 7. Further to evaluate the reliability of the developed mathematical model, two additional random compositions covering the entire range of experimental domains were prepared. For these additional formulations, the responses (Y_1_–Y_4_) were estimated with the help of generated mathematical models (Equations (3)–(5)). Finally, the experimental values obtained were compared with the expected or predicted values to calculate the bias (%) (Table 7). The lower bias is indicative of good agreement between the predicted and experimental values. Thus, bias is an index of high extrapolative ability and robustness of the quantitative mathematical models generated [15,61].

### 3.5. Evaluation of Efinaconazole Spanlastic Gel

#### 3.5.1. Topographic Imaging Using SEM

SEM was carried out to see the shape of spanlastics and their entrapment in the porous hydrogel structure of carbopol.

**Figure 4:**
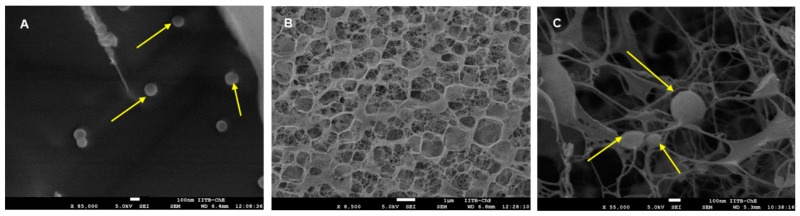
Representative scanning electron microscope images of (**A**) efinaconazole-loaded spanlastic vesicles, (**B**) blank carbopol gel, and (**C**) efinaconazole-loaded spanlastic vesicles loaded into carbopol gel for transungual delivery. Arrows indicate spanlastic vesicles.

Uniform spherical nanovesicles of efinaconazole-loaded spanlastic are seen in Figure 4A. The porous hydrogel structure of carbopol gel and efinaconazole-loaded spanlastic vesicles entrapped in porous carbopol hydrogel are seen in Figure 4B,C.

#### 3.5.2. Ex Vivo Permeation through Bovine Hoof Membranes

**Figure 5:**
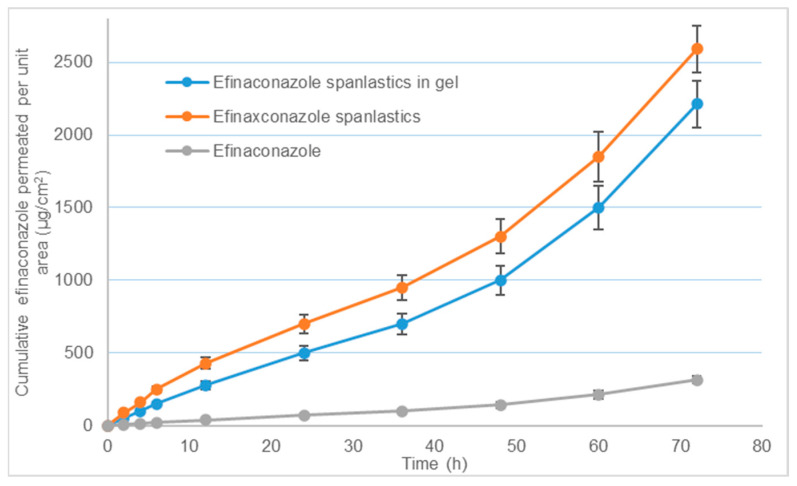
Release of efinaconazole vs. time profiles of pristine drug dispersion, spanlastics, and spanlastics dispersed in carbopol gel. Plotted values are the mean of six determinations and error bars represent standard deviation.

Bovine hoof membranes are commonly used to study nail delivery as their structure resembles the human nail [23]. The cumulative amount of efinaconazole permeated in 72 h from spanlastics and spanlastics dispersed in the gel were 2591 ± 162 and 2211 ± 158 μg/cm^2^, respectively (n = 6). The comparative permeation profiles are shown in Figure 5.

It can be observed that, in the same period, efinaconazole dispersion in water could permeate only 315.9 ± 22.6 μg/cm^2^ (n = 6). This indicates that spanlastic has significantly improved the penetration across the nail barrier. When compared between the two spanlastic formulations, gel showed slightly lower permeation due to a highly ordered hydrogel structure that restricted the mobility of nanovesicles within the gel structure resulting in the sustained release. Due to the adhesive nature of the gel, longer residence time over the nail surface will help prolong the duration of action, thus improving the efficacy.

## 4. Conclusions

This study systematically explained the need for the core regulatory application of a multifaceted techniques approach to understanding the behavior of formulation and process variables using FbD for the development of spanlastic nanovesicles of efinaconazole. Plackett–Burman screening design and full factorial design involving three factors at two levels as tools for designing the product were successfully utilized. With the help of Bayesian analysis, Lenth’s method, and mathematical modeling, formulation and process factors that were active in the quality features of the product were identified and optimized. Vesicle builder, Span 65, and the edge activator, Tween 80 were the most significant formulation parameters affecting the physicochemical properties of efinaconazole spanlastics. Within the considered experimental domain mixing time was also found to affect the quality of spanlastics. FbD using these three factors at two levels resulted in efinaconazole-loaded spanlastic vesicles with particle size; of 197 nm, transparency; 91%, relative deformability; 12.50 min, and dissolution efficiency; 81.23%. The mathematical model developed and validated had a high prediction power, showing FbD as an efficient tool in optimizing the composition and manufacturing process. This study also signifies that spanlastic nanovesicles represent an adaptable platform technology that can be optimized for a variety of poorly water-soluble drugs for delivery across tough biological membranes. The clinical applications of these easily-scaled-up and economically affordable nanovesicles can be expanded for other antifungal drugs for ungual and transungual delivery.

## Figures and Tables

**Table 1:** Various CFAs and CPPs named as X_1_–X_8_ along with their levels selected for 12-run PBSD analysis and the measured critical quality attributes (CQA; dependent variables Y_1_–Y_5_).

Parameter	CFAs and CPPs	Low Level (−1)	High Level (+1)
X1	Type of vesicle builder	Span 65	Span 60
X2	Type of edge activator	Sodium deoxycholate	Tween 80
X3	Mixing time (min)	10	20
X4	Amount of organic phase (mL)	5	10
X5	Sonication time (min)	0	5
X6	Amount of efinaconazole	10	15
X7	Mixing speed (rpm)	50	100
X8	Volume of aqueous phase (mL)	50	100
	CQA	Unit	
Y1	Particle size	nm	
Y2	Zeta potential	mV	
Y3	Relative deformability	min	
Y4	Transmittance	%	
Y5	Encapsulation efficiency	%	

**Table 2:** The layout of efinaconazole spanlastics displaying the levels of CFAs and CPPs along with observed CQAs (mean ± SD) for all 12 formulation batches of PBSD.

Batch	CFAs and CPPs Selected for Efinaconazole Spanlastics								CQAs				
	X_1_	X_2_	X_3_ (min)	X_4_ (mL)	X_5_ (min)	X_6_ (mg)	X_7_ (rpm)	X_8_ (mL)	Y_1_ (nm)	Y_2_ (mV)	Y_3_ (min)	Y_4_ (%)	Y_5_ (%)
P_1_	1	1	−1	1	1	1	1	−1	252.1 ± 4.1	−16.5 ± 0.5	16.8 ± 1.2	84.8 ± 2.1	63.1 ± 1.8
P_2_	−1	1	1	−1	1	1	1	−1	309.2 ± 3.7	−17.8 ± 0.7	30.6 ± 1.8	49.1 ± 1.2	65.2 ± 2.2
P_3_	1	−1	1	1	−1	1	1	1	778.3 ± 6.5	−14.3 ± 0.5	41.9 ± 2.3	32.2 ± 1.1	45.5 ± 1.8
P_4_	−1	1	−1	1	1	−1	1	1	313.2 ± 3.2	−16.7 ± 0.4	49.9 ± 3.6	68.7 ± 2.2	65.3 ± 3.1
P_5_	−1	−1	1	−1	1	1	−1	1	734.1 ± 6.0	−15.9 ± 0.4	48.9 ± 3.1	36.6 ± 1.2	55.9 ± 1.6
P_6_	−1	−1	−1	1	−1	1	1	−1	851.3 ± 4.9	−18.8 ± 0.6	56.7 ± 4.2	24.9 ± 0.9	81.9 ± 0.8
P_7_	1	−1	−1	−1	1	−1	1	1	613.3 ± 3.8	−16.2 ± 0.3	55.9 ± 2.2	48.7 ± 1.1	79.8 ± 1.2
P_8_	1	1	−1	−1	−1	1	−1	−1	411.2 ± 2.9	−18.3 ± 0.4	21.4 ± 1.8	68.9 ± 1.3	44.6 ± 2.1
P_9_	1	1	1	−1	−1	−1	1	−1	423.6 ± 3.9	−19.3 ± 0.4	25.2 ± 0.8	67.7 ± 1.7	27.9 ± 2.3
P_10_	−1	1	1	1	−1	−1	−1	1	479.2 ± 5.1	−18.1 ± 0.2	31.9 ± 1.1	42.1 ± 0.8	27.1 ± 1.6
P_11_	1	−1	1	1	1	−1	−1	−1	706.5 ± 6.3	−16.1 ± 0.4	47.1 ± 1.9	39.4 ± 1.5	55.8 ± 1.6
P_12_	−1	−1	−1	−1	−1	−1	−1	−1	862.2 ± 5.8	−14.9 ± 0.2	57.5 ± 2.0	23.8 ± 0.9	68.3 ± 1.4

**Table 3:** 2^3^ Full Factorial Design used in the experiment.

CFAs and CPP Were Used in 2^3^ Factorial Design	Levels of CFAs and CPP	
	−1	+1
X1: Amount of vesicle builder, Span 60 (mg/mL)	100	120
X2: Amount of edge activator, Tween 80 (mg/mL)	80	100
X3: Sonication time (min)	0	5
Unchanged variables levels		
Type of edge activator	Tween 80	
Type of Vesicle builder	Span 60	
Mixing time	10 min	
Mixing speed	50 rpm	
Amount of organic phase	10 mL	
Amount of aqueous phase	100 mL	
Amount of efinaconazole	15 mg	

**Table 4:** 2^3^ FbD. The eight formulation batches and their results mean particle size of spanlastics (Y_1_, nm), relative deformability (Y_2_, min), transmittance (Y_3_, %), and dissolution efficiency (Y_4_, %).

	CFAs and CPP			CQAs			
Formulation Run	X_1_	X_2_	X_3_ (min)	Y_1_ (nm)	Y_2_ (min)	Y_3_ (%)	Y_4_ (%)
FD1	−1	−1	−1	235.3	15.7	86.5	60.5
FD2	1	−1	−1	602.2	40.1	49.8	57.1
FD3	−1	1	−1	241.4	16.1	85.9	74.5
FD4	1	1	−1	569.7	37.9	53.1	71.0
FD5	−1	−1	1	241.3	16.1	85.9	62.5
FD6	1	−1	1	393.1	26.2	70.7	64.4
FD7	−1	1	1	180.5	12.0	92.0	81.3
FD8	1	1	1	302.9	20.2	79.8	85.4

**Table 5:** Various statistical parameters were derived and analysis of variance (ANOVA) for the 12 runs of PBSD was employed for eight independent variables for particle size (Y_1_), zeta potential (Y_2_), relative deformability (Y_3_), transmittance (Y_4_), and encapsulation efficiency (Y_5_). Significant values are in bold type.

Independent Variable	Coefficient	Particle Size		ZETA Potential		Relative Deformability		Transmittance		Encapsulation Efficiency	
		Coefficient	*p* Value	Coefficient	*p* Value	Coefficient	*p* Value	Coefficient	*p* Value	Coefficient	*p* Value
Constant	b0	560.92	**<0.0001**	−14.91	**<0.001**	40.32	**<0.0001**	48.91	**0.0003**	56.7	**<0.001**
Type of vesicle builder	b1	−30.42	**0.023**	0.125	0.84	−0.56	**0.009**	8.042	0.051	−3.92	0.10
Type of edge activator	b2	−196.42	**0.001**	−0.88	0.23	−11.02	**0.001**	14.64	**0.011**	−7.83	**0.018**
Mixing time	b3	10.58	0.233	−0.00	0.99	−2.72	0.065	−4.392	0.184	−10.47	**0.0081**
Amount of organic phase	b4	2.25	0.77	0.16	0.81	0.40	0.703	−0.225	0.935	−0.25	0.89
Sonication time	b5	−73.08	**0.002**	0.38	0.58	1.22	0.292	5.642	0.114	7.483	**0.021**
Amount of efinaconazole	b6	−5.08	−0.52	−0.03	0.97	−4.27	**0.021**	0.508	0.855	2.667	0.208
Mixing speed	b7	−13.08	0.16	−0.28	0.68	3.05	**0.049**	−0.358	0.897	4.233	0.084
Volume of aqueous phase	b8	−6.25	0.44	0.33	0.63	1.33	0.26	0.625	0.823	−3.667	0.115
**Statistical analysis of the model**											
Model *p* value		**0.0012**		**0.008**		**0.0107**		**0.007**		**0.034**	
F value		111.9		38.7		26.2		16.3		12.66	
Regression coefficient (r^2^)		0.99		0.90		0.99		0.94		0.971	

**Table 6:** Coefficients and their significance for CQAs: Y_1_ (particle size), Y_2_ (relative deformability), Y_3_ (transmittance), and Y_4_ (dissolution efficiency). Significant values are in bold type.

		*p* Values for Coefficients			
Factor	Coefficient	Y_1_	Y_2_	Y_3_	Y_4_
Intercept	b0	**0.0021**	**0.0024**	**0.001**	**0.0026**
X_1_	b1	**0.0060**	**0.0069**	**0.0059**	0.763
X_2_	b2	**0.0330**	**0.0374**	**0.0320**	**0.0216**
X_3_	b3	**0.0110**	**0.0126**	**0.0108**	**0.0479**
X_1_X_2_	b12	0.086	0.098	0.083	0.529
X_2_X_3_	b23	**0.0139**	**0.0160**	**0.0136**	0.112
X_1_X_3_	b13	**0.0469**	0.054	**0.0458**	0.122

Significant values are in bold type.

**Table 7:** The validation batches and the prediction error calculated as bias ^a^ (%).

Response	Y_1_			Y_2_			Y_3_			Y_4_		
Composition	Experimental Value	Predicted Value	Bias (%) ^a^	Experimental Value	Predicted Value	Bias (%) ^a^	Experimental Value	Predicted Value	Bias (%) ^a^	Experimental Value	Predicted Value	Bias (%) ^a^
A (105, 100, 5) ^b^	197.00	196.23	(+)0.39	12.50	13.09	(−)4.51	91.00	90.19	(+)0.90	81.23	82.03	(−)0.97
B (115, 85, 5) ^b^	330	335.01	(−)1.50	21.00	22.31	(−)5.89	75.00	76.69	(−)2.20	70.25	69.11	(+)1.65
C (107.5, 82.5, 2.5) ^b^	310	309.64	(+)0.12	20.00	20.65	(−)3.15	80.21	79.02	(+)1.51	65.12	64.46	(+)1.02

^a^ Bias = ((predicted -experimental)/experimental) × 100. ^b^ Values of amount of vesicle builder—Span 60 (mg/mL), amount of edge activator—Tween 80 (mg/mL), and sonication time (min).

## Data Availability

The data presented in this study are contained within the article.
